# Radiomic features of glucose metabolism enable prediction of outcome in mantle cell lymphoma

**DOI:** 10.1007/s00259-019-04420-6

**Published:** 2019-07-08

**Authors:** Marius E. Mayerhoefer, Christopher C. Riedl, Anita Kumar, Peter Gibbs, Michael Weber, Ilan Tal, Juliana Schilksy, Heiko Schöder

**Affiliations:** 1 Department of Radiology, Molecular Imaging and Therapy Service, Memorial Sloan Kettering Cancer Center New York, 1275 York Ave, New York, NY 10065 USA; 2 Department of Biomedical Imaging and Image-Guided Therapy, Medical University of Vienna, Vienna, Austria; 3 Department of Medicine, Lymphoma Service, Memorial Sloan Kettering Cancer Center New York, New York, NY USA; 4 Haifa, Israel

**Keywords:** Lymphoma, FDG, PET/CT, Prognosis

## Abstract

**Purpose:**

To determine whether [^18^F]FDG PET/CT-derived radiomic features alone or in combination with clinical, laboratory and biological parameters are predictive of 2-year progression-free survival (PFS) in patients with mantle cell lymphoma (MCL), and whether they enable outcome prognostication.

**Methods:**

Included in this retrospective study were 107 treatment-naive MCL patients scheduled to receive CD20 antibody-based immuno(chemo)therapy. Standardized uptake values (SUV), total lesion glycolysis, and 16 co-occurrence matrix radiomic features were extracted from metabolic tumour volumes on pretherapy [^18^F]FDG PET/CT scans. A multilayer perceptron neural network in combination with logistic regression analyses for feature selection was used for prediction of 2-year PFS. International prognostic indices for MCL (MIPI and MIPI-b) were calculated and combined with the radiomic data. Kaplan–Meier estimates with log-rank tests were used for PFS prognostication.

**Results:**

SUVmean (OR 1.272, *P =* 0.013) and Entropy (heterogeneity of glucose metabolism; OR 1.131, *P =* 0.027) were significantly predictive of 2-year PFS: median areas under the curve were 0.72 based on the two radiomic features alone, and 0.82 with the addition of clinical/laboratory/biological data. Higher SUVmean in combination with higher Entropy (SUVmean >3.55 and entropy >3.5), reflecting high “metabolic risk”, was associated with a poorer prognosis (median PFS 20.3 vs. 39.4 months, HR 2.285, *P* = 0.005). The best PFS prognostication was achieved using the MIPI-bm (MIPI-b and metabolic risk combined): median PFS 43.2, 38.2 and 20.3 months in the low-risk, intermediate-risk and high-risk groups respectively (*P* = 0.005).

**Conclusion:**

In MCL, the [^18^F]FDG PET/CT-derived radiomic features SUVmean and Entropy may improve prediction of 2-year PFS and PFS prognostication. The best results may be achieved using a combination of metabolic, clinical, laboratory and biological parameters.

## Introduction

Mantle cell lymphoma (MCL) is a rare subtype of B cell non-Hodgkin lymphoma, and can be associated with an aggressive or, less frequently, an indolent course [1]. Despite the availability of novel types of treatment, the prognosis in MCL patients is generally considered to be poor [2], with 5-year survival rates as low as 50% [1]. For estimation of prognosis, adapted versions of the International Prognostic Score (IPI) – the so-called MIPI scores, which incorporate age, ECOG performance status, leucocyte count, lactic dehydrogenase levels and in some variants also the Ki-67 proliferation index – are used in clinical practice [1]. These MIPI scores were built upon data for, and used for prediction of, 5-year survival, with a focus on overall survival (OS). No clinical, laboratory, or biological markers are currently established for prediction of shorter term clinical outcomes.

Pretherapy positron emission tomography/computed tomography after injection of the radiolabelled glucose analogue 2-^18^F-fluoro-2-deoxy-d-glucose ([^18^F]FDG PET/CT), which enables whole-body in vivo quantification of tumour glucose metabolism, has been shown to provide prognostic information in Hodgkin, diffuse large B cell (DLBCL), follicular and T cell lymphomas in a considerable number of studies, using quantitative parameters including the maximum standardized uptake value (SUVmax), total metabolic tumour volume (TMTV) and total lesion glycolysis (TLG) [3–9]. In MCL patients, only two studies have investigated the prognostic value of pretherapy SUVmax, with only one of these also including TMTV and TLG [10, 11]. The prognostic value of quantitative measures of [^18^F]FDG uptake heterogeneity across the TMTV, as can be provided by advanced radiomic analyses, have not so far been investigated in MCL patients.

Radiomics is an emerging field of research that is concerned with the computer-assisted extraction of quantitative, minable data from diagnostic medical images. Radiomic features include both traditional, first-order features (such as mean and maximum grey-level values), and more sophisticated features such as those that describe different aspects of image texture, which cannot be perceived by the human eye [12]. These image textural features have the potential to allow assessment of tumour heterogeneity [13, 14], which is recognized as a prognostic determinant of survival in different types of cancer [15–17]. Indeed, several studies in different types of cancer, and using different imaging techniques, have provided data that support the prognostic potential of radiomics [18–21], especially when processed by artificial intelligence-based machine-learning algorithms.

We therefore aimed to determine (1) whether [^18^F]FDG PET-derived radiomic features can predict 2-year progression-free survival (PFS), alone or in combination with clinical, laboratory and biological parameters, using a machine-learning algorithm, and (2) whether the [^18^F]FDG PET-based radiomic signature has prognostic value in comparison to, as well as in combination with, the established MIPI scores, in MCL patients receiving CD20 antibody-based immuno(chemo)therapy as first-line systemic treatment.

## Materials and methods

### Patients and design

Treatment-naive patients with histologically proven MCL (as diagnosed by a reference pathologist according to the current WHO classification), who had undergone [^18^F]FDG PET/CT for routine pretherapy staging at a single tertiary care centre between January 2010 and June 2016, were eligible for inclusion in this Health Insurance Portability and Accountability Act (HIPAA)-compliant, retrospective study. The study was approved by the Institutional Review Board of Memorial Sloan Kettering Cancer Center; informed consent was waived. Additional inclusion criteria were: documentation of clinical follow-up and imaging follow-up (by contrast-enhanced CT or [^18^F]FDG PET/CT) over a period of at least 2 years, or up to the date of death or progression within the 2-year observation period; clinical, laboratory and biological data, including ECOG performance status, white blood cell count (WBC), lactate dehydrogenase levels (LDH) and Ki-67 proliferation index, obtained within 1 week of the pretherapy PET/CT scan; and treatment with an R-CHOP-based regimen (rituximab, cyclophosphamide, doxorubicin, vincristine and prednisone, alone or in combination with high-dose cytarabine (HiDAC) and consecutive high-dose therapy and autologous stem cell therapy (HDT/ASCT) consolidation, or radioimmunotherapy with [^90^Y]-ibritumomab tiuxetan), or R-BENDA/O-BENDA (rituximab or ofatumumab, and bendamustine), or rituximab or ofatumumab monotherapy (in patients with low tumour burden). Patients with blood glucose levels >180 mg/dL and patients not examined with one of five prespecified PET/CT scanners (see below) were excluded.

### Imaging protocol

PET/CT covering the anatomy from the mid-skull to the upper thigh was performed approximately 60 min after intravenous administration of 12–15 mCi of [^18^F]FDG. Patients fasted for at least 6 h prior to [^18^F]FDG injection. PET was performed in three-dimensional (3D) mode, with at least 3 min per bed position, and a voxel size of 5.5 × 5.5 × 3.3 mm, using one of the following PET/CT scanners: Discovery ST, Discovery STE, Discovery 600, Discovery 690, or Discovery 710 (all manufactured by GE Healthcare, Waukesha, WI, USA). Spiral CT was performed with a tube current of 60 mAs, a tube voltage of 120–140 kVp, and a 5-mm section thickness, and was used for PET attenuation correction and anatomical correlation.

### Image analysis and radiomic signature

**Fig. 1:**
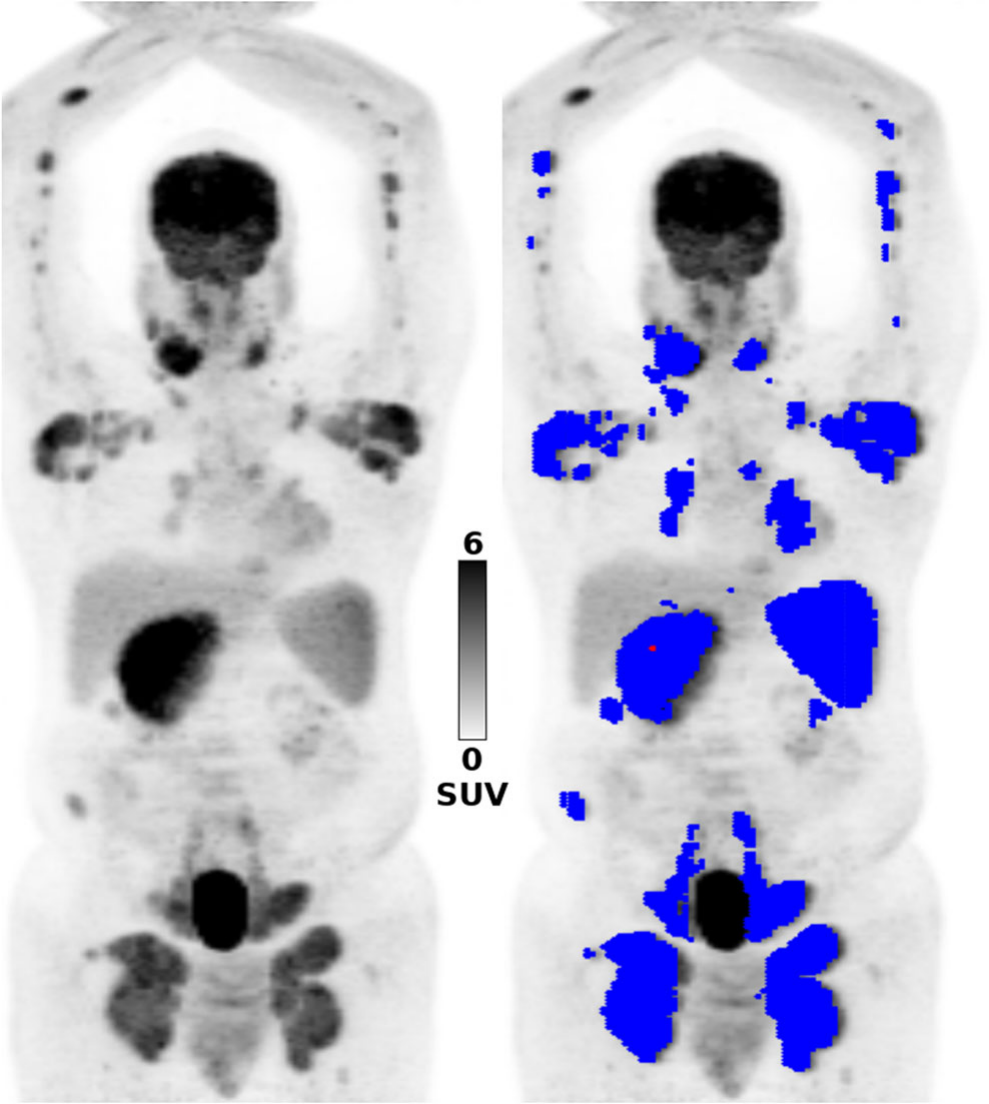
A 66-year-old patient with stage IV mantle cell lymphoma: *left* [^18^F]FDG PET maximum intensity projection image; *right* 3D radiomic analysis based on the total metabolic tumour volumes (*blue*) constructed using the previously recommended 41% SUVmax threshold; the SUVmax (*red dot*) was measured in the periportal nodal bulk

Using the Beth Israel PET/CT viewer plugin for FIJI [22], TMTVs were semiautomatically constructed, using the previously recommended 41% SUVmax threshold (Fig. 1) [23]. When there was low [^18^F]FDG uptake relative to the surrounding tissues, coregistered CT was used to aid manual lesion delineation. Based on the TMTVs, the SUVmax, SUVmean, SUVpeak and TLG (product of TMTV and SUVmean), as well as the following 16 textural features derived from the grey-level co-occurrence matrix were calculated in 3D: Entropy, Homogeneity, Contrast, Correlation, Angular second moment, Difference entropy, Difference variance, Inverse difference moment, Sum average, Sum entropy, Sum variance, Cluster prominence, Cluster shade, Maximum probability, and two Informational measures of correlation [24]. Equations for these textural features can be accessed at https://pyradiomics.readthedocs.io/en/latest/features.html. The 3D co-occurrence matrix was calculated with an interpixel distance of 1, and 13 directions; a minimum of 20 pixel pairs were used for each direction. Based on this, an arithmetic mean was calculated to provide a single value for each individual feature.

Radiomic features were harmonized using the previously described ComBat method to correct for technical differences between the PET/CT data from the different scanners. ComBat harmonization was originally described for use with genomic data and was subsequently validated for normalization of PET radiomic features so as to remove the centre effect while retaining the pathophysiological information [25]. This method is applied directly to the numerical values of the radiomic features, rather than to the PET images from which the radiomic features are calculated, and consequently, it does not lead to a reduction in the quality of subsets of images. ComBat-based transformations differ between the individual radiomic features obtained from each VOI and scanner, and are applied to the measured data so that they can be pooled without the need for separate training datasets [25].

Pearson and Kendall tau-b correlation coefficients were used, as appropriate, to evaluate the relationships between the different radiomic features, and also between radiomic features and overall PFS (in months) as well as 2-year PFS status, respectively. Univariate binary logistic regression analyses were used to identify radiomic features that were significantly predictive, at *P* ≤ 0.05, of 2-year PFS (i.e. 2-year PFS achieved or not). Based on radiomic features that were statistically significant in the univariate analysis, a multivariate logistic regression analysis with forward selection (based on likelihood ratio) was performed. Features that were significant in the multivariate analysis were regarded as representing the radiomic “signature” of MCL on [^18^F]FDG PET.

### Clinical data and MIPI score calculation

ECOG performance status, WBC, LDH level, Ki-67, Ann Arbor stage and blastoid differentiation (including blastic and pleomorphic variants) of the patients with MCL were recorded. Two established variants of the MIPI score were calculated, as previously described:

- The “classic” MIPI (based on age, ECOG performance status, WBC and LDH level) with three risk categories (high, intermediate, and low risk) [26]
- The “biological” MIPI-b (which also considers the Ki-67 index) with three risk categories (high, intermediate, and low risk) [26]

Simplified versions of the MIPI scores were not obtained. PFS was obtained from the electronic medical records of the hospital information system using the oncologist’s assessment and the original [^18^F]FDG PET/CT and CT reports. For PET/CT and CT, the Lugano response criteria for disease progression were applied [27].

### Machine learning for 2-year PFS prediction

A multilayer perceptron (MLP) feed-forward artificial neural network which relies on a back-propagation learning algorithm [28] was used to determine whether the [^18^F]FDG PET/CT radiomic signature can predict 2-year PFS. Because the starting point of the neural network is an initial guess at the weights of the individual radiomic features, the classification step was performed five times. The population of 107 patients was split into a training dataset and a validation dataset to which 70% and 30%, respectively, were randomly assigned; i.e. randomization of patients to the training and validation datasets differed for each repetition of the classification step. A minimum of one hidden layer (activation function: hyperbolic tangent), with a minimum of three neurons per hidden layer, was used for the MLP neural network (output activation function: softmax). Following the purely radiomics-based analysis described above, the classification step was repeated, again five times, this time using ECOG performance status, WBC, LDH level and Ki-67 index as additional input variables, to determine whether the integration of radiomic, clinical, laboratory and biological data could improve 2-year PFS prediction. Areas under the receiver operating characteristic (ROC) curves (AUCs) as well as classification accuracies for training and validation datasets were used as the main outcome measures.

### Radiomic signature and MIPI for PFS prognostication

Radiomic signature features were dichotomized using their respective ROC curve-based cut-off values. A single ordinal radiomic signature parameter reflecting the “metabolic risk” was then calculated using a “majority vote” system, with the categories “high metabolic risk” and “low metabolic risk”, and, in case of an even number of radiomic signature features, an additional “intermediate metabolic risk” category (same number of features above and below their respective cut-off values). Metabolic risk, as well as MIPI and MIPI-b were tested for PFS prognostication using Kaplan–Meier estimates, and the log-rank test was used for group comparisons. To determine whether the addition of metabolic risk improved MIPI risk categories in terms of PFS prognostication, the following strategy for MIPI and MIPI-b score modification was used:

- “High metabolic risk”: MIPI score +1, unless already highest score (i.e. MIPI or MIPI-b score 3, in which case MIPI score unmodified)
- “Low metabolic risk”: MIPI score −1, unless already lowest score (i.e. MIPI or MIPI-b score 1, in which case MIPI score unmodified)
- “Intermediate metabolic risk” (if applicable): MIPI score unmodified

Based on these modified MIPI and MIPI-b scores (termed “MIPI-m” and “MIPI-bm”), which included information about the metabolic risk, Kaplan–Meier estimates and log-rank tests were again performed. All statistical tests were performed using SPSS 24.0 (IBM Corp., Armonk, NY, USA).

## Results

**Fig. 2:**
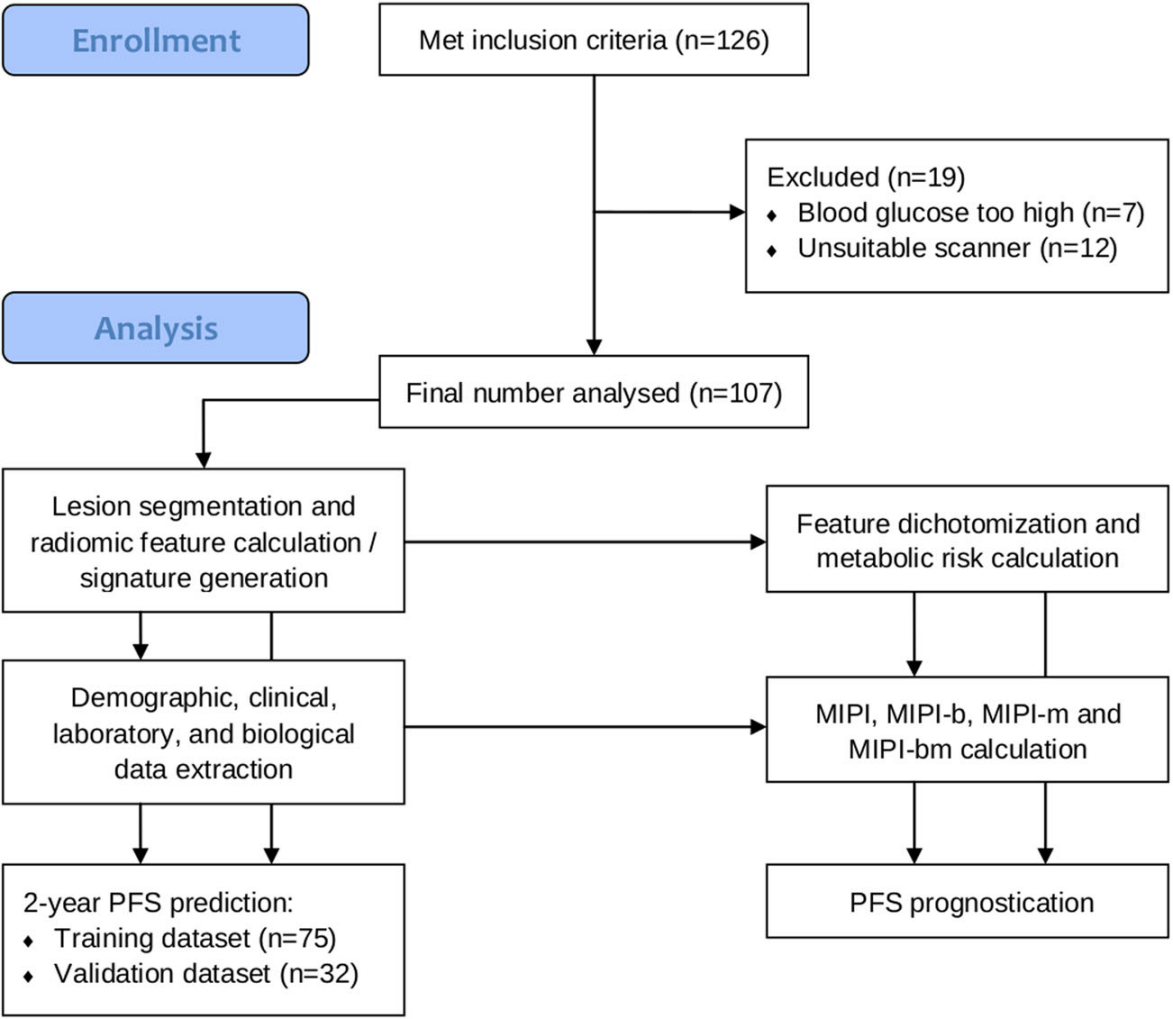
CONSORT diagram

A total of 107 consecutive patients (35 women and 72 men; mean age 64.5 ± 10.8 years) met the criteria for participation in the study (Fig. 2). [^18^F]FDG PET/CT was performed using the Discovery STE scanner in 43 patients, the Discovery 690 scanner in 41 patients, the Discovery 600 and 710 scanners in 10 patients each, and the Discovery ST scanner in 3 patients. Of the 107 patients, 58 were treated with an R-CHOP-based regimen (with additional HiDAC and HDT/ASCT in 42 patients, of whom 10 also received radioimmunotherapy), 38 with R-BENDA or O-BENDA, and 11 with rituximab or ofatumumab monotherapy. After 2 years, progression had occurred in 35 of the107 patients (37.2%), including 8 who had died. The median follow-up period was 52.6 months from registration, the median PFS was 50.4 months, and the median time to progression was 16.3 months. The patients’ baseline clinical, laboratory and biological characteristics are provided in Table 1.

**Table 1:** Baseline demographic, clinical, laboratory and biological data of 107 MCL patients, and the results of binary logistic regression analyses for continuous and categorical data

Characteristic	Frequency	Univariate analysis for 2-year PFS	
		OR (95% CI)	*P* value
Age	–	1.000 (0.961–1.041)	1.0
≥65 years	51 (47.7%)	1.251 (0.557–2.810)	0.59
Ann Arbor stage	–	–	–
I	5 (4.7%)	1	–
II	13 (12.1%)	1.286 (0.158–10.450)	0.81
II	23 (21.5%)	0.417 (0.054–3.221)	0.40
IV	66 (61.7%)	0.750 (0.117–4.822)	0.76
Blastoid differentiation	20 (18.7%)	0.857 (0.298–2.462)	0.78
Blastic	18 (16.8%)	–	–
Pleormorphic	2 (1.9%)	–	–
WBC	–	1.017 (0.982–1.053)	0.35
Elevated	18 (16.8%)	1.837 (0.653–5.165)	0.25
Ki-67 index	–	1.007 (0.991–1.022)	0.39
≥30%	56 (52.3%)	0.890 (0.396–2.001)	0.78
LDH level	–	1.004 (0.999–1.009)	0.096
Elevated	29 (27.1%)	1.116 (0.453–2.748)	0.81
ECOG performance status	–	–	–
≥2	7 (6.5%)	1.594 (0.337–7.545)	0.56

*OR* odds radio, *CI* confidence interval

### Radiomic signature

While radiomic features were not significantly correlated with absolute PFS (in months), 2-year PFS status was significantly correlated with SUVmean (*r* = 0.21, *P* = 0.008), SUVpeak (*r* = 0.19, *P* = 0.015), SUVmax (*r* = 0.19, *P* = 0.018), Entropy (*r* = 0.20, *P* = 0.012), Angular second moment (*r* = 0.18, *P* = 0.042) and Sum entropy (*r* = 0.17, *P* = 0.033). SUVmean (odds ratio, OR, 1.272, 95% confidence interval, CI, 1.037–1.560; *P =* 0.021) and Entropy (OR 5.070, 95% CI 1.156–22.234; *P =* 0.031) were the only radiomic features that were significantly predictive of 2-year PFS in the univariate analysis. Both SUVmean and Entropy, which did not show a significant correlation with each other (*r* = 0.17, *P* = 0.077), retained their statistical significance in the multivariate analysis (*P* = 0.022 and *P* = 0.034, respectively). Mean SUVmean was 3.78 (range 0.95–14.49) and mean Entropy was 3.48 (range 2.35–4.13). ROC analyses revealed an optimal SUVmean cut-off value of 3.55, and an optimal Entropy cut-off value of 3.5.

### Machine learning for 2-year PFS outcome prediction

**Fig. 3:**
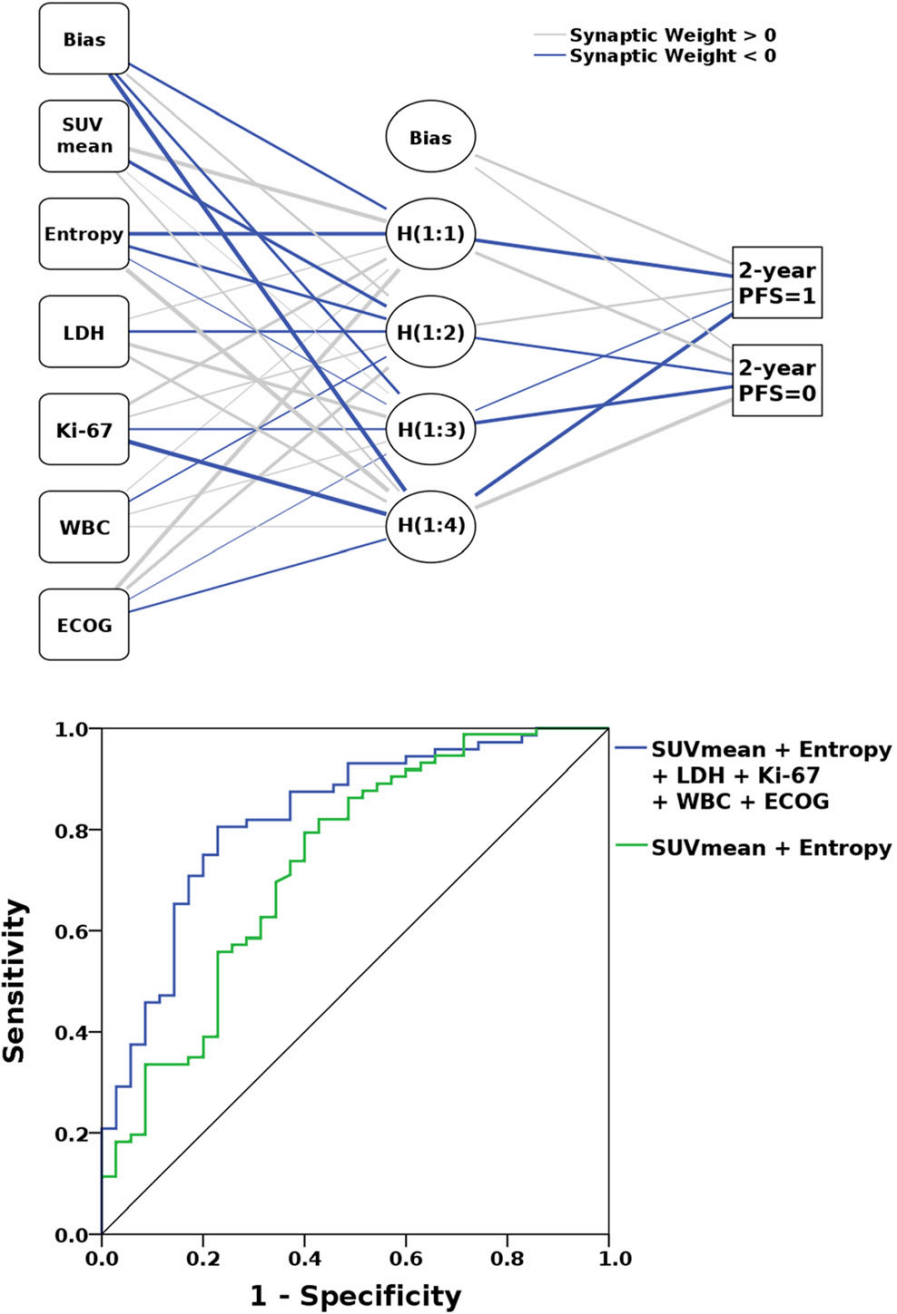
Results of the multi-layer perceptron (MLP) neural network-based prediction of 2-year PFS. With a single “hidden” layer with four neurons (*top* H(1:1) to H(1:4)), and SUVmean, Entropy, lactate dehydrogenase level (LDH), white blood count (WBC), Ki-67 index and ECOG performance status as inputs, the receiver operating characteristic (ROC) curve (*bottom*) yielded an area under the curve (AUC) of 0.83, whereas the use of just the two radiomic features (SUVmean and Entropy) as input for the neural network yielded an AUC of 0.73

With absolute SUVmean and Entropy values as input for the neural network, AUCs for 2-year PFS prediction were 0.70–0.73 (median 0.72); classification accuracies were 71.0–76.7% (median 74.4%) in the training dataset and 70.6–86.8% (median 74.3%) in the validation dataset. When, in addition to SUVmean and Entropy, ECOG performance status, WBC, LDH level and Ki-67 were added as inputs for the neural network, AUCs for 2-year PFS prediction were 0.77–0.83 (median 0.82; Fig. 3); here, classification accuracies were 72.5–82.9% (median 79.2%) in the training dataset, and 69.2–84.0% (median 76.7%) in the validation dataset.

### Radiomic signature and MIPI for PFS prognostication

**Fig. 4:**
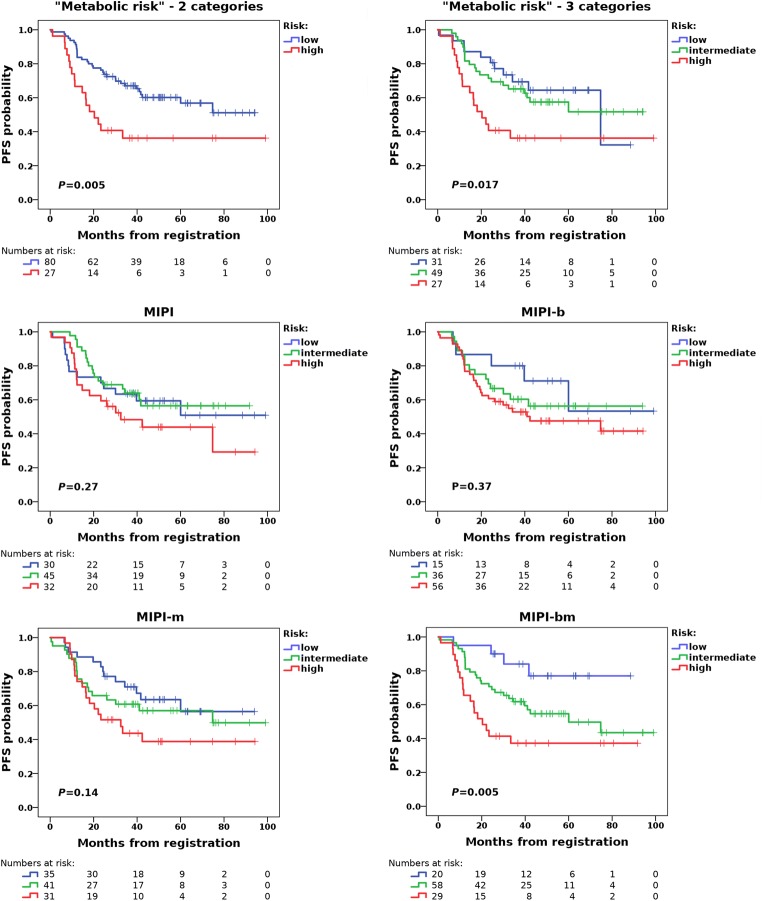
Kaplan–Meier estimates and log-rank tests show that the [^18^F]FDG PET/CT radiomic signature (combination of SUVmean and Entropy), which reflects “metabolic risk”, enables PFS prognostication. MIPI and MIPI-b are clearly improved by combining with metabolic risk, as assessed on [^18^F]FDG PET/CT. The best results are achieved with MIPI-bm (i.e. combination of MIPI-b and metabolic risk)

The [^18^F]FDG PET radiomic signature, consisting of dichotomized SUVmax and Entropy, was used to construct a two-category and a three-category prognostic model of metabolic risk for progression. In the two-category model, patients with high metabolic risk (SUVmean >3.55 and Entropy >3.5) had a median PFS of 20.3 months, with a 2-year PFS of 40.7% (11/27 patients), whereas patients with low metabolic risk (SUVmean ≤3.55 and/or Entropy ≤3.5) had a median PFS of 39.4 months, with a 2-year PFS of 76.3% (61/80 patients). PFS differed significantly between the two groups (*P* = 0.005; Table 2, Fig. 4). In the three-category model, patients with high metabolic risk (SUVmean >3.55 and Entropy >3.5) had a median PFS of 20.3 months, with a 2-year PFS of 40.7% (11/27 patients), patients with intermediate metabolic risk (SUVmean ≤3.55 or Entropy ≤3.5) had a median PFS of 40.3 months, with a 2-year PFS of 71.4% (35/49 patients), and patients with low metabolic risk (SUVmean ≤3.55 and Entropy ≤3.5) had a median PFS of 38.2 months, with a 2-year PFS of 83.9% (26/31 patients). PFS differed significantly among the three groups (*P* = 0.017; Table 2, Fig. 4).

**Table 2:** Descriptive data and results of log-rank tests for MIPIs, with and without modification due to “metabolic risk” on [^18^F]FDG PET/CT, for 107 MCL patients

Characteristic	Frequency	Median PFS (months)	Hazard radio (95% CI)	*P* value
Metabolic risk^a^ – two category model	–	–	–	0.005
Low risk	80 (74.8%)	20.3	1	–
High risk	27 (25.2%)	39.4	2.285 (1.264–4.131)	0.005
Metabolic risk^a^ – three category model	–	–	–	0.017
Low risk	31 (29.0%)	38.2	1	–
Intermediate risk	49 (45.8%)	40.3	1.225 (0.590–2.543)	0.59
High risk	27 (25.2%)	20.3	2.597 (1.212–5.564)	0.14
MIPI	–	–	–	0.27
Low risk	30 (28.0%)	41.7	1	–
Intermediate risk	45 (42.1%)	38.1	0.866 (0.424–1.771)	0.69
High risk	32 (29.9%)	27.7	1.463 (0.715–2.992)	0.30
MIPI-b	–	–	–	0.37
Low risk	15 (14.0%)	43.8	1	–
Intermediate risk	36 (33.6%)	35.7	1.441 (0.523–3.969)	0.48
High risk	56 (52.3%)	32.0	1.872 (0.724–4.843)	0.20
MIPI-m^b^	–	–	–	0.14
Low risk	35 (32.7%)	41.7	1	–
Intermediate risk	41 (38.3%)	37.6	1.344 (0.657–2.750)	0.42
High risk	31 (29.0%)	26.6	2.013 (0.984–4.120)	0.055
MIPI-bm^b^	–	–	–	0.005
Low risk	20 (18.7%)	43.2	1	–
Intermediate risk	58 (54.2%)	38.2	2.675 (0.935–7.653)	0.066
High risk	29 (27.1%)	20.3	4.884 (1.647–14.607)	0.004

*HR* hazard radio, relative to low risk group, *CI*confidence interval

^a^Based on the radiomic signature (combination of dichotomized SUVmean and Entropy)

^b^Modified according to metabolic risk

Both metabolic risk scores (i.e. with two and three risk categories) were superior to MIPI and MIPI-b (Table 2, Fig. 4). However, the best PFS prognostication with three risk categories was achieved with MIPI-bm (*P* = 0.005), i.e. the MIPI-b modified using the three metabolic risk categories (Table 2, Fig. 4).

## Discussion

Our study identified two main image-based predictors of 2-year PFS in MCL patients receiving CD20 antibody-based immuno(chemo)therapy: the average glucose metabolism across the entire MTV, as reflected by the SUVmean on pretherapy [^18^F]FDG PET/CT scans, and the heterogeneity of glucose metabolism within this MTV, as reflected by Entropy. The combination of the two radiomic features – i.e. the [^18^F]FDG PET radiomic signature – may also be useful for outcome prediction: higher SUVmean and higher Entropy appear to be associated with a shorter PFS (Fig. 4).

The three “metabolic risk” categories based on the radiomic signature were superior to the MIPI risk categories in terms of PFS prognostication, regardless of whether or not the Ki-67 proliferation index was considered in the calculation of the MIPI (Table 2, Fig. 4). The performance of the MIPI scores is not necessarily surprising, as OS, and not PFS, was the primary endpoint used in the development of MIPI and MIPI-b [26]. Notably, both MIPI and MIPI-b scores were considerably improved through combination with the [^18^F]FDG PET-based metabolic risk. In particular, unlike the MIPI-b, the MIPI-bm not only emerged as a statistically significant predictor of PFS, but was superior to metabolic risk alone (Table 2, Fig. 4), indicating that the best results may be achieved when clinical, laboratory and biological, as well as metabolic information, are integrated in a single model. This is also supported by the results of our machine-learning experiment, in which the addition of ECOG performance status, WBC, LDH level and Ki-67 index to the radiomic features clearly improved 2-year PFS prediction (Fig. 3). Since [^18^F]FDG PET/CT is currently recommended for staging and treatment response assessment in patients with MCL by the International Conference on Malignant Lymphoma (ICML) [27] – i.e. it is considered a standard procedure in these patients – information on the metabolic risk represents routine data, which may facilitate its integration into risk assessment in clinical practice.

The prognostic value of pretherapy quantitative [^18^F]FDG PET/CT in treatment-naive MCL patients has, to our knowledge, only been investigated in two prior studies. In a series of 81 patients, Karam et al. used the SUVmax to identify groups of MCL patients at risk of shorter survival [10]. The design of their study differed from ours in several ways. First, the SUVmax, which provides information about the single voxel with the highest glucose metabolism within the tumour volume, was the only [^18^F]FDG PET-based parameter evaluated, whereas radiomic analysis as performed in our study captures multiple facets of glucose metabolism across the entire metabolic lymphoma volume. Second, patients in the study by Karam et al. were chiefly treated with single-agent or combination chemotherapy (e.g. chlorambucil or CHOP), which does not reflect the present-day therapeutic state-of-the-art for first-line systemic treatment in MCL [29]. Third, three different PET scanners were used, but no correction was performed for the technical differences. Finally, while Karam et al. were able to successfully identify two MCL risk categories using an SUVmax cut-off value of 5, a further subdivision of MCLs with SUV >5 failed; no combination with MIPI scores or individual clinical/laboratory/biological data was attempted. In the second, very recent study, Albano et al. retrospectively evaluated three quantitative [^18^F]FDG PET/CT parameters – baseline SUVmax, TMTV and TLG – for outcome prediction in 87 MCL patients, using two different PET/CT scanners, also without applying correction for possible technical differences. These authors found that TMTV and TLG, but not SUVmax, were significantly associated with PFS using two risk categories. Contrary to our own study, neither SUVmean nor radiomic textural features reflecting the heterogeneity of glucose metabolism across the entire tumour, were included in their analysis, and no comparison or combination with MIPI scores was performed [11].

Entropy, a radiomic textural feature derived from the co-occurrence matrix, describes the degree of randomness, or disorder, in the distribution of image voxel grey-level values. Moon et al. recently demonstrated that [^18^F]FDG PET/CT-derived Entropy is correlated with the genetic heterogeneity index in lung cancer [13], whereas Choi et al. found that dual-energy CT-derived radiomic features, including Entropy, are also strongly correlated with the pathological heterogeneity index in lung cancer [14]. Entropy extracted from [^18^F]FDG PET/CT has recently been used for PFS prediction in patients with high-risk squamous cell carcinoma of the oropharynx after chemoradiation [30], and in patients with lung cancer after EGFR tyrosine kinase inhibitor treatment [31], and has also been found to be associated with failure to respond to third-line systemic treatment in metastatic colorectal cancer [32]. In lymphoma, however, pretherapy [^18^F]FDG PET/CT-based Entropy has so far been evaluated for prediction of interim response (i.e. the outcome after two therapy cycles) in paediatric Hodgkin lymphoma [33], and for prediction of disease-free survival and OS in aggressive B cell non-Hodgkin lymphoma (predominantly DLBCL, with a follow-up period of 3–54 months) [34], but did not emerge as a statistically significant marker in either study. Our study is therefore the first to show the value of Entropy on [^18^F]FDG PET/CT for outcome prediction in patients with a distinct histological lymphoma subtype, a finding that could be due to the fact that MCL is a “genomically unstable” tumour that may be associated with marked (sub)clonal heterogeneity, and also with heterogeneity between different topographic sites in the same patient, and with modulation of the initial mutational profile during disease progression [35].

We used PFS instead of OS as the clinical endpoint, which is contrary to many lymphoma studies investigating prognostic features. This strategy was chosen because MCL is a less common lymphoma subtype, and hence smaller patient populations and shorter follow-up periods may have to be used to obtain a sufficiently large number of cases with the event of interest. While OS is the established outcome, PFS has been recognized by the Food and Drug Administration as a valid surrogate endpoint in MCL and other haematological malignancies [36]. In a recent analysis of multiple randomized trials in DLBCL, including a total of 7,507 patients, PFS was significantly correlated with OS, supporting its use as a surrogate marker (https://www.fda.gov/Drugs/DevelopmentApprovalProcess/DevelopmentResources/ucm613636.htm).

Our study had some limitations. The most obvious limitations were the retrospective design and the modest cohort size. However, this was a hypothesis-generating study, as [^18^F]FDG PET-derived radiomic features have not been previously evaluated for outcome prediction and prognostication in MCL patients. Furthermore, with a sample size of 107 patients, this is the largest study on this topic at present. Our approach involving the combination of radiomic features and MIPI scores – i.e. using the three categories of metabolic risk to modify MIPI scores – was exploratory. However, MIPI scores were clearly improved by applying this strategy, and in addition, the combination of the (continuous) radiomic feature values and the clinical, laboratory and biological parameters (which were also used for calculation of the MIPI scores), using a machine-learning algorithm, showed the same trend: best results were achieved when all parameters were integrated into a single model. We used an MLP neural network for outcome prediction, which is a universal function approximator with the ability to model any type of regression or classification problem [37]. While MLP networks are well established in the machine-learning community as powerful prediction algorithms [38], it is possible that even more advanced, deep machine-learning techniques such as convolutional neural networks (CNN), with their larger numbers of hidden layers and their interconnection between neurons within the same layer, may have performed even better. However, CNNs are mainly intended for use with large datasets (“big data”). In a patient population such as our own, however, their complexity would have increased the probability of “overfitting”, i.e. loss of generalizability of the model, in our case for PFS prediction [39].

In conclusion, an [^18^F]FDG PET radiomic signature comprising SUVmean and Entropy has prognostic value in MCL and may be useful for predicting early tumour progression. This metabolic risk reflected by radiomic features can be integrated into MIPI scores and may possibly improve risk stratification in MCL. Further studies are warranted to validate these findings in external cohorts.
